# Antimicrobial resistance in a war-torn country: Lessons learned in the Eastern Democratic Republic of the Congo

**DOI:** 10.1016/j.onehlt.2019.100120

**Published:** 2019-12-16

**Authors:** Gabriel Kambale Bunduki, Jean-Louis Mumbere Katembo, Ildéfonse Soly Kamwira

**Affiliations:** a Department of Infectious diseases, Faculty of Medicine, Université Catholique du Graben, Butembo, Democratic Republic of the Congo; b Association for Health Innovation in Africa, Butembo, Democratic Republic of the Congo; c Department of Anatomo-Pathology, Cliniques Universitaires du Graben, Faculty of Medicine, Université Catholique du Graben, Butembo, Democratic Republic of the Congo; d Faculty of Pharmaceutical sciences, Université Catholique du Graben, Butembo, Democratic Republic of the Congo

**Keywords:** Antimicrobial resistance, National action plans, Surveillance, Laboratory, War-torn, DRC

## Abstract

There is no doubt that antibiotics have reduced the burden of bacterial infectious diseases. Antimicrobial resistance threatens the effectiveness of successful treatment of infections and constitutes a public health concern with national and global dimensions. This problem is worrisome in war-torn areas like the Eastern Democratic Republic of the Congo. The difficulties experienced by war-torn nations in addressing antimicrobial resistance are significant for the rest of the globe as microbes do not acknowledge boundaries and do not distinguish between peace and wartime. In this paper, we describe the impact of warfare on the social determinants of health, the environment and biodiversity, and its consequences on the antibiotic use and the host-pathogen interplay. Furthermore, we describe different pillars to be taken into account, learned in a war-torn area, in combating antimicrobial resistance. These lessons are summarized in terms of tools to be used for combating antimicrobial resistance, challenges to overcome in war-torn setting and core actions to be undertaken. Surveillance is a valuable tool to combat antimicrobial resistance as it helps to detect resistant bacteria, enables correct decisions to be taken, guides policy recommendations and tracks the antibiotic use and misuse. The challenges encountered in this region include the shortage of competent laboratories, poor infrastructure and data management, lack of standard protocols, low coverage of surveillance, lack of intersectoral cooperation, and inadequate national, regional and international collaboration. Regarding this situation, the core actions to be undertaken include the establishment of ABR surveillance and monitoring systems, building laboratory capacity for rapid and reliable diagnostic testing, and engagement in national, regional and global surveillance networks. Therefore, this study showed an urgent need for establishing and implementing sentinel site surveillance laboratories and elaborating and implementing national action plans for combating antimicrobial resistance.

## 1 Introduction

Currently, there is no doubt that antibiotics have reduced the burden of bacterial infectious diseases. Millions of people might have died due to these infections in the absence of antibiotics [1]. Unfortunately, antimicrobial resistance (AMR) threatens the effectiveness of successful treatment of infections and constitutes a public health concern of national and global dimensions [2]. AMR is an ongoing phenomenon observed in bacteria, and its spread and amplification is accelerated by irrational use of antibiotics, the use of fake and counterfeit drugs, poor prescribing habits and non-compliance to prescribed treatments [3]. In the Eastern part of the Democratic Republic of the Congo (DRC), a region that has been facing war, conflicts and an humanitarian crisis; this situation have been fuelled by several factors.

The difficulties experienced by war-torn nations in addressing AMR are significant for the rest of the globe as microbes do not ‘acknowledge’ boundaries and do not ‘distinguish’ between peace and wartime. In addition, nations typically divert resources in times of conflict to satisfy military requirements rather than the population's health needs. These unfortunate conditions provide an atmosphere for bacteria to establish mechanisms of resistance to traditional antibiotics, as well as opportunities for the spread of emerging pathogens.

In the Eastern DRC, the health system has been disrupted and weakened, supply shortages in health facilities, poor or scarce availability of microbiological surveillance and high antimicrobial resistance has been reported [4,5]. In this paper we will argue on the impact of warfare on the social determinants of health, the environment and biodiversity, and its consequences on the antibiotic use and the host-pathogen interplay. In addition to that, we will describe different pillars for combating antimicrobial resistance. These pillars will be summarized into three major ones: (1) surveillance as a tool to combat AMR, (2) challenges to overcome in war-torn setting and (3) core actions to undertake.

## 2 Impact of warfare on the social determinants of health, the environment and biodiversity, and on antibiotic use

The social determinants of health are “the circumstances, in which people are born, grow up, live, work and age, and the systems put in place to deal with illness. These circumstances are in turn shaped by a wider set of forces: economics, social policies, and politics” [6]. Conflict exacerbates existing health inequalities. Due to the challenges of working in conflict contexts, there is a lack of evidence to support best practices in emergency response and particularly a lack of evidence on the application of social determinants in these settings [6,7].

Direct measures are the most common during a conflict and in post-conflict impact analysis [7]. These include mortality, access to care, medication access, and sanitation coverage. Indirect indicators include DALYS (Disability Adjusted Life Years), morbidity, specifically emergency related infectious diseases such as cholera, Ebola, malaria, shigellosis, measles as well as a complementary child centered indicators [7]. In a conflict setting, the direct impact on health is due to death, injuries or disabilities caused by violence. Indirect impacts are associated with conflict as a result of the complete disruption of daily social life and infrastructure. The morbidity and mortality that is indirectly associated with conflict is difficult to identify and to disentangle from those due to other underlying causes of poor health [7]. More deaths in Eastern DRC are due to conditions of displacement that lead to diseases. The Eastern DRC has long records of poor health outcomes due to the combined impact of conflict, poverty and ineffective health systems [8].

The three determinants of health that the World Health Organization (WHO) found in all conflict settings include the loss of human rights, breaches of medical neutrality, and the progression from individual “stress to distress to disease” due to recurrent exposure to violence [9].

In conflict settings, the loss of the human rights is most acutely expressed as lack of security, displacement, loss of social networks and family structure that comprise the fabric and meaning of daily life and its social reciprocities, loss of livelihood and loss of daily activity, access to land and employment opportunities that provide for daily life and needs resulting in extreme poverty, food insecurity due to loss of land and resources for livelihood, lack of shelter, lack of safe water and sanitation, lack of essential health and other social services, such as education for children, and lack of communication, leading to isolation [9].

Breaches of medical neutrality comprise a second social determinant specific to a conflict setting. These are especially relevant for the right to health care, as combatants attempt to weaken the resistance of civilians by deliberately depriving them of access to care, especially at the times when they most need it. A few examples of violations include attacks on health facilities and staff, attacks on health care providers and patients, attacks on medical convoys and ambulances, barriers, checkpoints and barrier walls that obstruct access to care, the politicization of health services, resulting in discrimination in the provision of health care on the basis of social identity. In Eastern DRC, many other breaches of medical neutrality have been recorded. Armed men have entered hospitals, demanded treatment for their injured comrades or have randomly attacked health staff. Like teachers and university professors, health staffs are captured for ransom; they are assumed to belong to families and/or corporations that can pay the ransom and are therefore targeted [9].

When warfare causes the mass movement of people, the resulting impacts on the environment can be catastrophic. Widespread deforestation, unchecked hunting, soil erosion, and contamination of land and water by human waste occur when thousands of humans are forced to settle in a new area [10]. Recent research points to the environment as an important component for the transmission of resistant bacteria and in the emergence of resistant pathogens. Apart from transmission between humans, environmental dissemination routes for resistant bacteria have also been pointed out as potentially important for the spread of antimicrobial resistance. Environments facilitating dissemination of resistant bacteria also enable spread of non-resistant human pathogens, and generally also opportunistic pathogens. Thus, sewage, wastewater treatment plants, water bodies and travel, but also air-borne aerosols, dust, and food colonized by bacteria, are important vectors enabling bacterial transmission between hosts through the environment [11].

War is an ever-present force that has the potential to alter the biosphere. Dramatic habitat alteration, environmental pollution, and disturbance contributed to population declines and biodiversity losses arising from both acute and chronic effects in both terrestrial and aquatic systems. Armed conflicts found within terrestrial ecosystems often facilitates poaching by military forces and can promote further destruction of the landscape and wildlife populations by displaced refugees of war. The weapons employed by militaries probably pose the greatest hazard by terrestrial conflicts to ecosystem structure. The numerous explosive techniques and tools at the disposal of army forces during ground warfare have left a legacy on landscapes across the globe by leaving large craters, shrapnel, and contamination, thus devastating many ecosystems across the biosphere. The impacts of conflict, nuclear weapons, training operations, and chemical contaminations all contribute to both reductions in the populations of local flora and fauna as well as reducing species diversity in the affected ecosystems [12].

With war, trauma, communicable diseases related to morbidity and mortality as well as wound infections has become particularly public health concern. A war wound has an enormous potential for bacterial infection and hence requires the use of antibiotics. This relates not only to the direct victims of military conflict but also to the displaced civilians. Since in Eastern DRC dispensing of antibiotics without appropriate prescription is common [3], such practice may lead to the spread of antimicrobial resistance among the local bacteria frequently causing both community-acquired and nosocomial infections. Practice of self-diagnosis and self-medication with antibiotics by patients themselves and restraint to pharmacist advice is widespread in the region.

Fluctuating environments can modulate host–pathogen interactions by providing a temporary advantage to one of the interacting organisms. Human infections caused by highly drug-resistant pathogens are more prolonged, complicated, and difficult to eradicate [13].

## 3 Different pillars to consider for combating AMR

### 3.1 Pillar 1: surveillance as a tool to combat AMR

Surveillance is a useful tool to combat AMR as it helps (a) to detect resistant bacteria, (b) to enable correct decisions to be taken, (c) to guide policy recommendations and (d) track antibiotic use and misuse.

Surveillance is a tool used to follow the spread of resistant bacteria in the population or in particular geographic areas, and help in the prompt notification and investigation of drug-resistant diseases outbreaks. Findings from surveillance will give the right information on the therapeutic actions to be undertaken and will guide policy recommendations and the monitoring of prevention and control measures applied to combat the infection [1]. The use and misuse of antibiotics should be monitored to assessing the public health consequences [3].

Surveillance involves the collection and analysis of data for the detection and monitoring of threats to public health. Surveillance should also inform as to the epidemiology of the threat and its burden in the population. A further key component of surveillance is the timely feedback of data to stakeholders with a view to generating action aimed at reducing or preventing the public health threat being monitored. Surveillance of antimicrobial resistance involves the collection of antibiotic susceptibility test results undertaken by microbiology laboratories on bacteria isolated from clinical samples sent for investigation. Correlation of these data with demographic and clinical data for the patient populations from whom the pathogens were isolated gives insight into the underlying epidemiology and facilitates the formulation of rational interventions aimed at reducing the burden of resistance [14].

The DRC has no global national coordination of AMR. Only a few organisms are targeted and are subject to surveillance. Among them, tuberculosis drug resistance and typhoid fever due to *Salmonella typhi* have been monitored for several years. The Eastern DRC has been served by medical laboratories of low to medium quality. Most of the tests performed by these laboratories are only for therapeutic aims but not for surveillance. Few laboratories in either private or public sector are accredited.

For surveillance of antimicrobial resistance in DRC, the essential core data might be generated by microbiology laboratories that routinely identify and determine the susceptibility or resistance of bacteria isolated from clinical specimens. These results might be stored in the laboratory computer system and if accessed, collected and analysed, could inform as to the degree of antibiotic resistance seen in different bacterial species or isolates from different types of infection. Changes or variation in antibiotic resistance either geographically or over time could also be monitored. Proper identification of AMR requires verification of strains by molecular typing methods. Typing is the characterization of isolates or strains below species or subspecies level. These are useful for long-term surveillance as these methods give definite results. Molecular typing makes it possible to track the dissemination of specific clones; it may facilitate the breaking down of endemic transmission to the level of micro-epidemics [15].In the genomic era, the introduction of molecular methods has largely replaced phenotypic methods and “molecular epidemiology” has emerged as a new discipline. The current molecular typing methods include PCR-based methods, DNA fragment analysis-based methods, and DNA sequence-based methods, including the new exciting era of high-throughput genome sequencing [16]. As there is a lack of equipped laboratories in war-torn settings, isolates of interest may be sent to a reference laboratory for strain verification.

The voluntary reporting of microbiological diagnoses by hospital laboratories to DRC Ministry of Health (MoH) may be a mainstay of infectious disease surveillance in DRC. The report can be done on paper forms or electronically. This surveillance system can be strengthening by including a wide geographical coverage, the large amount of data collected and by the fact that the data may be made readily available on a continuous basis as routine outputs from laboratories. In addition to the collection of routinely generated laboratory results, surveillance systems may also be established by involving the collection of bacterial isolates from sentinel laboratories for testing in a centralized facility, typically a national Reference Laboratory. Establishing and implementing sentinel laboratories in each province of DRC may contribute in collecting AMR data from the whole country and increasing the amount of data collected. These laboratories will also constitute a national network for AMR surveillance.

### 3.2 Pillar 2: challenges to overcome in war-torn setting

Although efforts have been done in gathering and using AMR data in tuberculosis and salmonellosis, challenges still remain. Major challenges include the shortage of competent laboratories, poor infrastructure and data management, lack of standard protocols, low surveillance coverage, lack of intersectoral cooperation, and inadequate national, regional and international collaboration.

The AMR surveillance depends on microbiological laboratories quality to accurately identify resistant bacteria [17]. Generally, the Eastern DRC lacks such laboratories and in the few places where they exist, the means for quality control are often lacking. The lack of laboratory based-surveillance precludes the timely diagnosis of emerging infectious diseases. The ongoing Ebola Virus Disease (EVD) outbreak is more illustrative. In early May 2018, a disease of unknown aetiology was responsible for deaths of people in the rural commune of Mangina. Due to lack of local laboratory for surveillance, samples were sent to the Institut National de Recherches Biomédicales (INRB) located in the capital city, Kinshasa, in late July; and Ebola virus was confirmed to be the cause of those deaths. A recent research reported a typhoid fever case with a *Salmonella enterica* serovar Typhi isolate showing extended spectrum β-lactamase (ESBL) production in DRC [18]. This case was suspected with typhoid fever in the health zone of Panzi in Kwango. The blood sample was collected for culture and shipped to the INRB in Kinshasa. Upon arrival, there was growth of Gram negative rods, which were identified as *Salmonella* Typhi by standard biochemical reactions. The isolate was shipped to Belgium for further investigation for which the whole genome sequencing revealed that the strain carried a plasmid-mediated CTX-M-15 ESBL gene and did not belong to the dominant H58 *Salmonella* Typhi clade. This finding of ESBL production in *Salmonella* Typhi in DRC is of huge concern, as it adds to the high proportion of multidrug resistance [18]. If such strain spreads all over the country, this may be an outbreak of major concern. These few example show the importance of implementing sentinel surveillance laboratory well equipped in each province of the country.

In DRC, diseases with epidemics potential are under surveillance. These include cholera (*Vibrio cholerae*), Shigellosis (Shigella), tuberculosis, Measles, yellow fever, Chikungunya, dengue, typhoid fever (Salmonella typhi), meningococcal meningitis, and hemorrhagic fevers (Ebola, Marburg, rift valley, Crimea-Congo, West Nile). In addition to that, antimicrobial resistance surveillance is done for the following pathogens *Staphylococcus aureus*, enterococci, *Streptococcus pneumoniae*, *Escherichia coli*, *Klebsiella pneumoniae*, *Enterobacter* spp., *Pseudomonas aeruginosa* and Neisseria gonorrheae [19].

In the Eastern DRC, many hospitals have less than basic microbiology laboratory facilities. Some institutions have all the needed microbiologic resources, while others have none. Some hospital laboratories have instruments and reagents yet have no qualified technical staff to use them. Others may be able to amplify genomes yet cannot report the results of a simple Gram stain in a timely manner. It has been proven that laboratory medicine in developing countries has been severely neglected and has become a serious impediment to effective healthcare delivery and diseases surveillance. Physicians have little confidence in laboratory test results even if laboratory facilities exist, and rely on history-taking and physical examination for patient management. Hence, inadequate resources are allocated to laboratory services, which in turn results in less than optimal quality assured results, which leading to the neglect of laboratory services [20]. This situation leads also to poor data management which prevents routine monitoring and reliable data collection to measure the extent of AMR.

The lack of standard protocols in measuring resistance leads to variation in methods used; hence there are difficulties to compare data between laboratories in the country. The lack of intersectoral cooperation impedes the integration of the one health approach in the AMR surveillance. The impact on human health of using antibiotics as growth promoters and for disease prevention in food-producing animals is not studied at all. This cannot be evaluated without a perfect collaboration for AMR surveillance of pathogens from humans, food products and animals.

### 3.3 Pillar 3: core actions to undertake

Core actions to be undertaken for combating antibiotic resistance have been formulated by the World Health Organization (WHO) in terms of the WHO policy package to combat antimicrobial resistance [21]. Specific core actions to be undertaken in the African region have also been described previously [22]. Here, we summarize them in 3 actions: (a) establishment of AMR surveillance and monitoring systems, (b) building laboratory capacity for rapid and reliable diagnostic testing, and (c) engagement in national, regional and global surveillance networks.

The surveillance and monitoring of AMR can be done by establishing a national action plan which will bring together all the required recommended measures, stakeholders of public health, policy makers and partners. This will help in reducing the emergence of resistant pathogens and their spread. The surveillance and monitoring of AMR can be sample-based, establishment of sentinel site surveillance, use of standardized protocols, and the use of software and information system like the WHONET. Therefore, pathogens to be monitored should be prioritized based on the country diseases burden and antibiotics selected to be tested for each pathogen should take into account the list of essential medicines used in the country and treatment guidelines.

The laboratory capacity and its reliability for testing are useful in combating AMR. The country should designate and capacitate in equipment and human resources some laboratories as national reference laboratory. In addition to that, for achieving the reliability of testing, the establishment of quality assurance system in these laboratories will help in generating adequate and reliable AMR data which will guide actions to be taken for combating AMR. In addition to internal quality assurance done regularly on reagents and tests in these laboratories, they should also be participating in external quality assurance done at national, regional or international level. Achieving all these conditions in war-torn settings is challenging. The government may strengthen the existing laboratories in equipment and human resources. One to two laboratories may be chosen in each of the 26 provinces of the DRC and be capacitating as reference laboratory. This one may then supervised other laboratories within the province for quality assessment.

Networks in combating AMR will help in sharing information regarding AMR surveillance in many countries of a region. This is useful when taking decision at a country and regional level. This is also important in the case of outbreaks of pathogens of public health concern. Therefore, networks should be built in national, regional and global levels [23]. Combating AMR within a surveillance network increases the number of pathogens surveyed in that particular region and allow discriminating more trends and outbreaks earlier. It also decreases blind spots where new resistance clones may emerge and spread unseen. In contrast, past resistance clones may have spread widely in regions where laboratories are sparse before being noticed.

## 4 Conclusion

The situation of antibiotic resistance surveillance in the Eastern Democratic Republic of the Congo depicts the situation of the whole country. Therefore, there is an urgent need for establishing and implementing sentinel site surveillance laboratories and elaborating and implementing national action plans for combating antibiotic resistance.

## Authors contributions

GKB participated in the conception and design of the study, literature search, and drafted the manuscript. JMK and SK participated in literature search. All authors read and approved the submitted manuscript.

## Declaration of Competing Interest

None declared.
